# The Medico-Chi Wins

**Published:** 1899-05

**Authors:** L. Webster Fox

**Affiliations:** Secretary of the Board of Trustees; Philadelphia


### THE MEDICO-CHI WINS.

PHILADELPHIA, March 22, 1899.

TO THE EDITOR:

SIR,—The Medico-Chirurgical College petitioned Common Pleas Court No. 3 for leave to amend its charter so as to grant diplomas and degrees in dental surgery, etc.

This was resisted by the Philadelphia Dental College on the ground of want of authority to do so, etc. The Common Pleas Court decided in favor of the Medico-Chi, and the Dental College took an appeal from his decision. The Supreme Court, in an opinion by Justice Dean, has just confirmed the decision of the lower court, and dismissed the appeal.

L. WEBSTER FOX, M.D.,
*Secretary of the Board of Trustees.*

